# Characterizing the Experiences and Educational Needs of Patients and Caregivers During the Kidney Transplant Process

**DOI:** 10.1177/20543581251399080

**Published:** 2025-12-25

**Authors:** Michelle Ruhl, Ashley Burghall, Brianna Groot, Nicola Rosaasen, Kayla Flood, Keefe Davis, Natasha Minakakis, Jenny Wichart, Holly Mansell

**Affiliations:** 1 Division of Pediatric Nephrology, Department of Pediatrics, Stollery Children’s Hospital, University of Alberta, Edmonton, Canada; 2 College of Pharmacy and Nutrition, University of Saskatchewan, Saskatoon, Canada; 3 Division of Pediatric Nephrology, Department of Pediatrics, Jim Pattison Children’s Hospital, University of Saskatchewan, Saskatoon, Canada; 4 Patient and Family Advisor, Saskatoon, SK, Canada; 5 Department of Pharmacy, Alberta Health Services, Calgary, Canada

**Keywords:** pediatrics, caregivers, patient education, perceptions, solid-organ transplantation

## Abstract

**Background:**

Kidney transplantation, a life-saving therapy for children with kidney disease, remains challenging to navigate for patients and families.

**Objective:**

To elucidate the experiences and educational needs of patients who have received a kidney transplant and their caregivers.

**Design:**

Qualitative descriptive study.

**Setting:**

One province in Canada (Saskatchewan)

**Patients:**

Patients who received a pediatric kidney transplant, transplant recipients and caregivers.

**Methods:**

Semi-structured interviews were conducted via video or by phone and recorded and transcribed verbatim in this qualitative descriptive study. Qualitative content analysis was used to analyze the data. Decontextualization involved an inductive approach, whereby the text was coded and organized into categories and subcategories. Dedoose^®^ software was used to facilitate this process.

**Results:**

Twenty-three individuals participated, including 13 caregivers (aging in range from 20’s to 60’s) and 10 patients who had previously received a transplant (aging in range from adolescents to 40’s). Three categories emerged from their experiences: (1) the impact of the transplant on the individual (subcategories social, mental health, physical, lifestyle, returning to normal and new life perspective); (2) transplant expectations (transplant as a cure, unexpected experiences); and (3) the need for support throughout the transplant process (practical support, mental health support, healthcare support, support through shared-lived experiences, and challenges related to finding a community). Regarding education, participants identified the need for personalized, age-appropriate education delivered in digestible formats, with clear expectations, timely reinforcement, and emotional support tailored to both patients and caregivers.

**Limitations:**

Participants were recruited from a single small center in Canada. Patient participants had received their transplant at least 5 years prior to participation in the study and were reflecting on their past experiences.

**Conclusion:**

Feeling prepared for the transplant journey impacts the transplant experience and kidney transplant education can facilitate care and clarify expectations. The insights gathered from the study will help inform the development of educational resources for patients and caregivers.

## Abrégé

**Contexte:**

La transplantation rénale, qui est une thérapie vitale pour les enfants atteints d’insuffisance rénale, reste un parcours difficile à traverser pour les patients et leurs familles.

**Objectif:**

Connaître les expériences vécues et les besoins éducatifs des patients transplantés du rein et de leurs aidants.

**Conception:**

Étude qualitative et descriptive.

**Cadre:**

Une province canadienne (Saskatchewan)

**Sujets:**

Des patients (adultes et d’âge pédiatrique) transplantés du rein alors qu’ils étaient enfants, ainsi que leurs aidants.

**Méthodologie:**

Des entretiens semi-structurés ont été menés par vidéo ou par téléphone, enregistrés et transcrits verbatim. Une analyze qualitative du contenu a servi pour l’analyze des données. La décontextualisation s’est appuyée sur une approche inductive, dans laquelle le texte a été codé puis regroupé en catégories et sous-catégories. Le logiciel Dedoose^®^ a été utilisé pour faciliter le processus.

**Résultats:**

Vingt-trois personnes ont participé, soit treize aidants (âgés de 20 à 60 ans) et dix patients receveurs d’une greffe (âgés de l’adolescence à 40 ans). Trois catégories de données sont ressorties de leurs expériences: 1) l’impact de la greffe sur l’individu (sous-catégories : vie sociale, santé mentale, santé physique, mode de vie, retour à la normale et nouvelle perspective de vie); 2) les attentes envers la transplantation (perception de la greffe comme guérison, expériences inattendues); et 3) le besoin de soutien tout au long du processus (soutien pratique, soutien en santé mentale, soutien médical, soutien par le partage d’expériences vécues et pour la recherche d’une communauté). Sur le plan éducatif, les participants ont souligné le besoin d’une éducation personnalisée et adaptée à l’âge, dispensée dans des formats compréhensibles et intégrant des attentes clairement définies, un renforcement en temps opportun et un soutien émotionnel ciblé, tant pour les patients que pour leurs aidants.

**Limites:**

Les participants ont été recrutés dans un seul center canadien de petite taille. Au moment de leur participation, les patients avaient reçu leur greffe depuis au moins 5 ans, leurs réponses concernaient donc leurs expériences passées.

**Conclusion:**

Être préparé au parcours de transplantation influence significativement l’expérience des patients. Une éducation appropriée en transplantation rénale permet de faciliter les soins et de clarifier les attentes. Les renseignements tirés de cette étude contribueront à guider l’élaboration de ressources éducatives destinées aux patients transplantés et à leurs aidants.

## Introduction

Kidney transplantation is considered the treatment of choice for children with kidney disease,^1^ but it can be extremely challenging for patients and caregivers. Families must navigate new and complex medical information throughout the transplant journey. Knowledge of the transplant process and what to expect before, during, and after the transplant, increases the likelihood of a successful transplant experience.^2^ Ideally, information is tailored to the patient’s and family’s level of medical literacy to optimize understanding. After transplant, patients must strictly adhere to a complex regimen of immunosuppressant medications, which expose the patient to new risks such as infections and malignancy. Understanding the timing, administration, and ongoing titration of transplant medications is critical for success.^3,4^ A lifelong commitment to lifestyle changes (e.g. hygiene practices and infection prevention) and new monitoring practices (e.g. routine bloodwork, medical appointments, and follow-up with the transplant team) is required. For pediatric patients, family caregivers provide much of this support and, in most circumstances, are required to attend appointments throughout the transplant journey until the child transitions into adulthood. Pediatric kidney transplantation is performed in specialized centers, adding complexity for families who must travel to obtain care. In our own institution (Saskatchewan, Canada), patients and caregivers are required to travel out of province for transplant surgery. Families relocate for 6 to 8 weeks until the recipient is stable enough to return to Saskatchewan and resume care at home. Previous work with caregivers of adult lung transplant recipients identified additional psychological, emotional, logistical, and financial burdens associated with traveling away from home for transplantation^5^ and stimulated the development of new resources to improve pre-transplant education for this population.^6^ Educational needs in the context of pediatric kidney transplantation are unknown and deserve to be explored.

Given the importance of transplant education, there is a surprising lack of research in this area. We conducted a scoping review to summarize existing literature and identified only 18 articles evaluating patient-focused educational interventions.^7^ In a secondary objective to describe the educational experiences and needs of patients and caregivers, participants indicated that the transplant process was stressful or overwhelming (n = 6/9 studies). They indicated that social supports and education helped them cope with the stress of transplant,^8-13^ highlighting the importance of transplant education. Throughout these reports, participants consistently wanted more information than they received.^7^ Notably, only 5 studies pertained to kidney transplant populations,^8,9,12,14,15^ and all except one report^9^ were at least a decade old. Moreover, all kidney transplant qualitative studies, except two,^12,14^ excluded patients, instead focusing exclusively on caregiver experience.

The purpose of this study was to conduct an exploration of the educational needs during the pediatric process by interviewing patients who have received a kidney transplant as a child as well as with caregivers. Our objectives were to characterize the transplant experiences of the participants and to learn about their educational requirements before and after a pediatric kidney transplant.

## Methods

### Study Population and Recruitment

The study was approved by the Behavioural Ethics Review Board at the University of Saskatchewan (Beh#3511). The participants were patients who had received a kidney transplant as a child and caregivers in one province in Canada. Such patients were identified through the pediatric kidney transplant program (patients and caregivers) and adult kidney transplant program (patients who were transplanted as children but have since transitioned to the adult program for follow-up care). Prospective participants were approached by their regular healthcare provider to determine initial interest, and for potential patients under the age of 18, the caregiver was informed about the study prior to a conversation with the child. Potential participants were told that participation was completely optional, and their care would not be affected either way. Those expressing support were contacted by the research team. Pediatric patients and caregivers were offered the opportunity to participate jointly or individually. All participants then received a detailed study information package and were scheduled for a recorded telephone or video interview at least 48 hours after informed consent/assent was provided. To preserve patient confidentiality, the care team was not informed of their study participation status.

### Data Collection

The questions and interview approach were designed by the research team in collaboration with a patient partner after an extensive review of the literature on pediatric transplant education.^7^ The final version included questions designed to explore their lived experiences, determine which information was perceived to be important throughout the stages of the transplant process,^12^ assess satisfaction with their educational experience, and tabulate suggested resources/supports for improvement (Supplementary Appendix A). Trained interviewers (i.e., a pharmacy student and a research associate with an MSc, both female) who were not known to the participants conducted the visits (BG and AB). Participants were told the rational for the study and the questions were asked one at a time until all open-ended questions were answered or until the participant chose to end the interview, whichever came first. Interviews were audio-recorded, and field notes were taken during the interview. To ensure transparency, participants were invited to review and edit their transcripts prior to analysis. All participants received $75 CAD or equivalent for their participation in this study.

### Data Analysis

Data was categorized by members of the research team (BG, AB, and HM) through qualitative content analysis^16^ to minimize abstraction and preserve participant input. The deidentified transcripts were divided among the group, and one reviewer led the first-pass analysis of each interview. Dedoose^®^ (version 9.0.78) qualitative software was used to facilitate the process. Decontextualization involved an inductive approach, whereby one reviewer analyzed the transcripts line by line. Meaning units were labeled with codes, which were further organized according to categories and subcategories. Descriptive analysis occurred at the manifest level with low abstraction and low interpretation,^17^ and the process was iterative. Group members reviewed the coding completed by each other and then met periodically to provide suggestions for category relabeling and refinement and come to consensus before proceeding with the next set of transcripts. After the coding was completed and the research team had achieved consensus on the categories, two researchers (HM and MR) drafted the results. The results were presented according to patient and caregiver subgroups. The decision was made to combine “adults who had been transplant as children” and “adolescents” into a single subgroup of patients since all patient participants had been transplanted at least 5 years ago (and in most cases more than 10) and to protect the anonymity of the three adolescents.

Several practices were used to establish trustworthiness of the data.^18^ An iterative process involving multiple coders and internal debriefing sessions was used to maintain credibility and confirmability. Participants were also provided with the opportunity to review and discuss the final manuscript prior to publication, and a patient and family advisor provided guidance throughout the research process. Supplementary quote tables from multiple patients and caregivers are included for each category to support transferability. To ensure dependability, we maintained a documented audit trail and shared research folders (which included iterations of the interview guide, notes from the interviews, team meetings and analytical process) and reported our results according to the Consolidated Criteria for Reporting Qualitative Research (COREQ).^19^

## Results

### Participants

Thirteen caregivers (including parents, a grandparent, and one spouse of a former pediatric patient who was not present during the pediatric transplant, aged 20’s to 60’s) and 10 patients who had previously received a kidney transplant participated in a one-time interview. Patients ranged from 13 to 42 years of age; however, all patients started their transplant process and received pre-transplant education before adulthood. All patient participants had received their transplant at least 5 years prior to participating in the study, while most caregivers (61%) had more recent transplant experience (less than 4 years post-transplant). Ninety percent (n = 21) of the participants identified as Caucasian. Demographic data are displayed in Table 1. The interviews ranged from 45 to 81 minutes; the average interview length was 61 minutes. A total of 16 interviews were conducted: 9 individual and 7 joint interviews. One interview was conducted by email as the participant requested accommodation. All interviews with adolescent participants (those aged 10-19) were conducted jointly with their caregivers.

**Table 1.** Self-Reported Characteristics of Study Participants (n = 23).

Characteristic	Patient (n = 10) number (%)	Caregiver (n = 13) number (%)
**Age of participant (years)**		
10-19	3 (30%)	-
20-39	5 (50%)	1 (8%)
40-59	2 (20%)	9 (69%)
60-69	-	1 (8%)
Unknown		2 (15%)
**Age of child at time of transplant (years)**		
0-4	2 (20%)	8 (61%)
5-9	4 (40%)	1 (8%)
10-14	1 (10%)	2 (15%)
15-19	3 (30%)	2 (15%)
**Time since transplant (years)**		
0-4	-	8 (61%)
5-9	1 (10%)	1 (8%)
10-14	2 (20%)	3 (23%)
15-19	2 (20%)	-
20-24	4 (40%)	1 (1%)
25-29	-	-
30-34	1 (10%)	-
**Sex/Gender**		
Male	5 (50%)/5 (50%)	4 (31%)/4 (31%)
Female	5 (50%)/5 (50%)	9 (69%)/9 (69%)
**Ethnicity** ^a^		
African	-	1 (8%)
White	9 (90%)	12 (92%)
Canadian Indigenous	-	1 (8%)
Southeast Asian	1 (10%)	-
**Location of residence**		
Urban	6 (60%)	5 (38%)
Rural	3 (30%)	8 (62%)
Urban and rural (shared custody)	1 (10%)	-
**Educational achievement**^b^		
Some elementary	1 (10%)	-
Completed elementary	2 (20%)	4 (31%)
Completed high school	3 (30%)	1 (8%)
Completed technical certificate	-	2 (15%)
Completed postsecondary	4 (40%)	6 (46%)

a Percentage will not add up to 100 since some participants stated more than one answer.

b Not all patients were an eligible age to attend.

### Objective 1. Exploring Transplant Experiences

**Figure 1.**
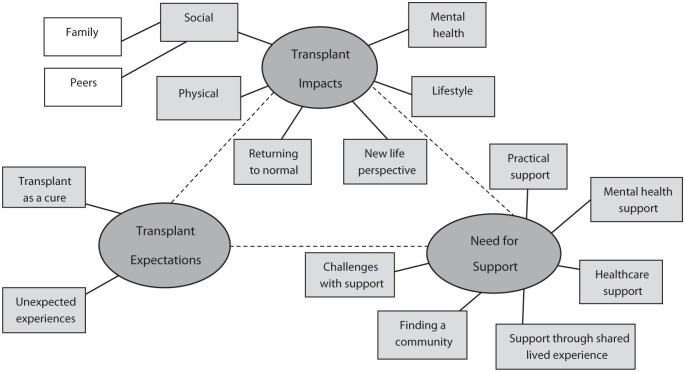
Overview of categories and subcategories pertaining to patient and caregiver transplant experiences.

Three overarching content categories were identified in the transcripts, including (1) transplant impacts (subcategories social, mental health, physical, lifestyle, returning to normal and new life perspective); (2) transplant expectations (transplant as a cure and unexpected experiences); and (3) the need for support throughout the transplant process (subcategories practical support, mental health support, healthcare support, support through shared-lived experience and challenges related to finding a community). (Figure 1). The categories are described in the following section, with additional supporting quotes available in Tables 2-4.

**Table 2.** Transplant Impacts: Additional Supporting Quotes.

Sub-category	Caregiver quotes	Patient quotes
Social	Transcript 21: *“We tried to keep as much of a normal life as we could for the older kids. They ended up having then to stay with family if we were away for appointments or surgeries.”* Transcript 10: *“Yeah, elementary school was really hard for him. He felt like an outsider. . . It was it was difficult for him to make a connection.”*	Transcript 20: *“I have a brother and a sister. . . oftentimes the other two feel neglected because they don’t get the same attention because they’re fine.”* Transcript 2: *“I would say it’s changed everything. My family dynamic. My childhood growing up. A lot of my childhood was spent with doctors or adults, so I didn’t have the same kind of interactions.”*
*Mental*	Transcript 21: *“I have a hard time remembering before the surgery because the emotions were so high. I think you need a good support team. The hardest part was, for us, his dad going into surgery, then him going into surgery, you’ve got so many worries back and forth you’re hoping that it works, it’s so long.”* Transcript 10: *“I had a depression shortly after I moved to Edmonton because I was very suddenly removed from home and family. . . I was following the book on how to be pregnant. I didn’t actually do anything wrong, but I felt like I grew him wrong, and I gave him this life. I had a lot of guilt.”*	Transcript 18: *“I probably came to terms with my mortality a little bit younger than a lot of other people so maybe that made me a bit more serious child than other kids, so less carefree in some respects.”* Transcript 1: *“When someone’s willing to put their life on the line for you, that’s a big deal. You have some very interesting places that your mind goes to, some unfortunately very dark places: “What if something happens to my family members when they’re trying to donate?” when they’re on the table — you get really weepy and emotional about that .”*
*Physical*	Transcript 11: *“He’s also had cancer twice. It’s a side effect of one of his medications. . .we knew it had a small chance of it happening, but we never thought that it would happen, and it happened twice.”* Transcript 6: *“I think you can’t stress enough how much a child changes after transplant. They’ll grow from their weight and their energy is just totally different. I think this summer that she had with her new kidney it made up for all the summers that she wasn’t feeling good.”*	Transcript 11: *“Yeah, the appointments and IVs sucks due to how small my veins are now it hurts, it hurts.”* Transcript 3: *“I was struggling to even just get through high school most days. Immediately after that transplant I had energy, I had an appetite, I felt amazing.”* Transcript 16: *“Yeah, acne [was an unexpected side-effect]. I never had acne in my life until I started taking ‘em pills. . .It’s minor, but it was major to me.”*
*Lifestyle*	Transcript 14: *“Well, there’s a lot of activities that she can’t partake per say, especially being young and just not have the ability to watch if something happens. We weren’t able to put her in a hockey or a net sport; she wasn’t able to do soccer. They really wanted her to be cautious of any kind of physical activity or sport.”* Transcript 14: *“The amount of appointments was extremely overwhelming, and I know that for myself, our situation, I had to quit my job because it was just inconvenient to be at work.”*	Transcript 18: *“It’s like first thing in the morning you get up, have breakfast, take your meds, and carry on with your day. And right before I go to bed, take meds, and that sort of thing. Right, it’s part of my morning routine, part of my going to bed routine, kind of like brushing your teeth.”*
*Returning to normal*	Transcript 21: *“You would almost not think that anything was wrong with him except for he takes a handful of pills in the morning.”* Transcript 6: *“It feels like she’s any other kid. Like we don’t feel like we have a kid with a chronic disease anymore at all.”*	Transcript 2: “*I would say just how much day-to-day life changed. . .Recess. Dietary. Playing with friends. Sleepovers. Just all that kind of stuff.”* Transcript 16: *“You just want to be normal. So, you just go on with everything that you can, unless something serious happens. But you just want to live a normal life.”*
*New Life Perspective*	Transcript 8: *“So, as Christians, we believe that whatever we do, God is with us. The doctor came the night before the surgery. . . he told us how many hours it’s going to be in the OR and he also told us that maybe 18% were not successful. . .I just rejected that 18%. I know it’s going to be successful for my son.*” Transcript 15: *“My husband and I talk a lot about how fortunate we are. . .There are many families that are dealing with way worse things than what we have or are. We have a whole lot more to be grateful for than we have to feel sorry for.”*	Transcript 4: *“I’m glad to have second chance at life after transplant.”* Transcript 18: *“It does give you a new lease on life for sure. I felt way better right after the transplant and my parents say I looked way better, like. as soon as I had the transplant so having a transplant can be is something hopeful to look up for most people.”*

**Table 3.** Transplant Expectations: Additional Supporting Quotes.

Sub-category	Caregiver quotes	Patient quotes
Transplant as a cure	Transcript 10: *“I kind of thought it would stabilize through life, and it would become something that was regular and normal. . . But even now I guess it feels like the next emergency. . . It’s always something new, like a learning curve.”* Transcript 6: *“One thing that always surprises me so much, I didn’t know was that I thought once you had a kidney, you’re good to go for the rest of your life. I didn’t know that you probably would have to get another one if you’re very young.”* Transcript 17: *“He has always known that he was gonna need a transplant. It was part of our discussion really his whole life, so he knew that was coming and he was okay with getting a transplant. It had it gone well, and he thought it was just going to, I guess, improve his quality of life and we heard that a lot. . .So he struggled a little bit with some of the sports they talked about restricting and with all the side effects.”* Transcript 21: *“I don’t think we prepared ourselves for complications,. We just thought we’re gonna get a kidney transplant, we’re gonna wait the three months that you have to sit by the hospital and then we’re gonna go home.”*	Transcript 3: *“As a teenager it was kinda frustrating that you had to [do frequent monitoring] again now you thought you were healthy.”* Transcript 1: *“They didn’t know if the kidney was going to make it, they didn’t know if I was going to make it. After the revision, there were even more complications, more surgeries. Which again, you can disclaimer that this is how it may go, this is how it may not go, and you just cannot plan for every single contingency. I wish that I had known that certain other things may happen.”* Transcript 20: *“I think that that’s a major part of letting them [patients and caregivers] know that it’s not going to necessarily be a miracle cure. I think that, not that anybody ever said that, I don’t think that that’s how they present it, but that’s your hope. Your hope is that all of these issues you’ve had are going to be behind you and it’s going to be clear sailing from here on out. . .so when you fit into that group it’s a bit more of a shock, even though you know about it”*
Unexpected experiences	Transcript 15: *“I think we knew there would be follow ups, but I don’t know that we realized that it would be a weekly blood draw initially. I understand the importance of that but on a two-year-old, that’s traumatizing.”* Transcript 6: *“I guess we kind of expected her [daughter] to be herself and talk as herself for the first week but that didn’t happen until three weeks that’s when she actually started smiling and being the [daughter] I knew.”* Transcript 17: *“I was shocked by what he looked like and everything we dealt with right after. I wasn’t expecting that.”* Transcript 10: *“So, I would say the psychology side of things is one that I wasn’t prepared for.”*	Transcript 3: *“We really kinda thought they would just test her [my loved one], she would be a match, or she wouldn’t, and then we would get done. So, yes that wait time was quite surprising that it took that long.”* Transcript 3: *“I got a phone call in July saying that there was a patient in the OR with a kidney that matched me and she was going in and if it didn’t work for her, then I would be the next one called so be prepared for that. You’re told not to get your hopes up, but just like, you have your hopes up. It’s kinda scary and at that time I didn’t realize. . . the kidney might not actually work, there might be other complications. That was also something that I learnt more as the process happened.”*

**Table 4.** Need for Support Additional Quotations.

Sub-category	Caregiver quotes	Patient quotes
*Practical support*	Transcript 14: *“If you and your husband are full-time workers, do you have another support system that can maybe help you get your child to appointments? We are lucky that we had that.”* Transcript 8: *“The social worker connected us to the Kinsmen Foundation and this foundation has been a great help. Supporting us with the accommodation and they feed him, it’s a lot of relief, so they really made it a lot easier for us.”* Transcript 6: *“Well, we had to make sure that somebody’s always available to give [daughter] her medication.”*	
*Mental health support*	Transcript 15: *“I have a friend. If I’m having a tearful day, she’s very good. She’s just like, ‘But you’re allowed. You’re allowed to feel how you’re feeling.’”* Transcript 16: *“And mental health support, because you can imagine there’s actually quite a lot of stress and fear that goes along with all of this, because there’s a lot of unknown and a lot of worries of something not working or something not happening, all that kind of stuff.”*	Transcript 1: *“That would be a really good place for social work or counseling, whether it’s spiritual, religious, holistic; having someone to talk to.”* Transcript 16: *“Yep. And if I had to be honest, it’s [mental health support] a humongous gap right now. . . Anytime we’ve reached out for mental health support. . .We’ve had to push really, really hard to get something that could even remotely be helpful.”*
*Healthcare* support	Transcript 8: *“I know the surgeon would come and check in while we were there almost every morning. And he’s still saying that he misses the surgeon.”* Transcript 10: *“The social workers at the X, they are angels. . .There’s like one person handling 15 families, I think. And she comes with her big heart of gold and her calmness and then she’s like, I know it’s a lot, but if you feel this in what we can do is get you a life supplement that can help you week by week with these expenses and Oh my goodness, social workers are unsung heroes.”* Transcript 15: *“Her team needs to be cloned. They are so supportive. That team at X—I think they love [patient] as much as we do, or they definitely display that. They are so there for us.”*	Transcript 3: *“I really liked meeting the transplant team and how they had a pharmacist on hand, they had a nurse on hand, and I really liked that I could discuss with them and I felt like they understood it better than the doctor, if you know what I mean. You could discuss more and then they could relate how you were feeling to the doctor, if you couldn’t put it into words that he would understand. I never felt like I was rushed with them, that they always had time if I needed to talk a bit more, they were there so that was really good. . . That was a huge thing was from my previous appointments with the nephrologist it was just my doctor that I talked to, right. Now I felt like I could call any time because I wouldn’t be bothering the doctor and I wouldn’t necessarily be bothering just one person, there was a team there, there’d always be someone available, so that was really nice.”*
*Support from shared-lived experience*	Transcript 6: *“The most helpful would have been to know that there are many kids that have received a transplant and that have had success with it. That we aren’t the only ones that are going through this. . .to be able to connect with people who have children who have gone through a kidney transplant.”*	Transcript 18: *“You feel like you’re a little bit different than other kids, so being able to meet another transplant patient at a young age would be [helpful]. Just someone to talk to would be handy as well.”* Transcript 4 (describing a camp they attended): *“I was about 9- or 10-years-old. . .It helped me meet others that had kidney disease and to understand more.”*
*Finding a community*	Transcript 15: *“I would say, get your little circle of caregivers, prior. You may not need them, but if nothing else, it’s. . .family members. . .some close friends, people that you trust. If you’re having a bad day, you could reach out to them and say, ‘I’m feeling discouraged today or I’m feeling tired.’ I think that would be so important. . .sometimes one just needs a little bit of moral support.”*	Transcript 18: *“My family already had a fairly big extended family so we kind of have a built-in support network in that respect and you know we had like school and church communities to fall back on which kind of helped.”* Transcript 3: *“We definitely benefitted from our church family and relatives and friends. Everybody was very supportive, and we lived in a small community, the community itself was very supportive too. We had a lot of support.”*
*Challenges with support*	Transcript 15: *“Maybe we should have. . .explained more to other family members. We could have had other family members want to be part of [patient]’s circle; part of her team. That would have helped. The more the merrier. Maybe we could have had some friends on board. Some people would be honoured. . .there are a lot of people who would love to learn about that. It’s important to not get frustrated with those who don’t, because it’s not for everybody. It doesn’t mean that they don’t love [patient] or don’t want to be part of her life, but they’re not prepared to take on.”*	Transcript 4; P1: *“I don’t think I had support before and after. No one knows what I was going through. . . I did wish I had support, but it is what it is.”*

### Category 1: Transplant Impacts

#### Social

Transplantation profoundly impacted the entire family. Both patients and caregivers described a pervasive feeling of social isolation. In the words of one patient (transcript 18), “*. . . you feel like kind of an exception. You feel like you’re a little bit different than other kids*.” Caregivers strived to maintain a sense of normalcy but were often challenged by the logistics of caring for a sick child. For example, one said (transcript 2),

*“My mom had to essentially just stop working so that she could be with me. And he [my Dad] would have to do weekend trips. . . we lived in the Ronald McDonald House in Edmonton and so I didn’t really have a sense of home for a while.”*

The impact on siblings was also noted. According to one caregiver (transcript 15), *“The other kids—children are so resilient. They need little crowns as well, her brother and sister, because they were so accepting. . .They never once resented her [the transplant recipient].”*

Children undergoing the transplant process often struggled with fitting in with their peers and readjusting socially after the transplant. One patient (transcript 1) said: *“I basically lost a year of school, year and a half maybe? I did not get to graduate with my friends. It’s a tough social reintegration almost. People sometimes don’t know how to treat you when you come back.”* Lifestyle restrictions often caused a sense of “missing out” For example, a caregiver from transcript 21 explained,

*“She’s kind of missed out on that team sport togetherness and having friends in that type of thing. Like she only has friends at school, and she’s never got to experience a team gathering, so maybe that was hard on her.”*

#### Mental health

Mental health impacts were common as well. From a caregiver perspective, many aspects of the transplant process were described as stressful, including approaching a relative about organ donation, fearing that they personally wouldn’t be eligible to donate their kidney, guilt for having a sick child, and post-transplant concern for their child’s health, even when it was stable. One caregiver (transcript 15) said, *“I’m always thinking the worst. What if she injures that kidney? She only has one and her daddy can’t give her his other one.”*

Mental health struggles were described by both patients and caregivers. One caregiver said (transcript 10),

*“[Patient] entered into a state of depression and self-loathing. . .And I didn’t picture that that would come, but he has had a lot in his life to challenge him. So, of course he got run down. He looks at his body after he’s had prednisone infusions and he’s puffed up and he gets zits, and it feels ugly and I just don’t like that he saw himself that way. . .So, I would say the psychology side of things is one that I wasn’t prepared for.”*

A sense of resilience was also described. According to a caregiver from transcript 6, *“Well, I think it kind of made us very strong. It made us feel that we can go through tough times. Sometimes you have to actually go through things to see what you’re capable of doing.”*

#### Physical

Participants described the remarkable physical impacts from the kidney transplant, including a return of energy, appetite, and overall health. Caregiver from transcript 10:

*“He got an appetite for the first time in his life. I think it was three days post-transplant and he sat himself up in bed and reached for a grilled cheese sandwich. That was astounding. His cheeks and his feet turned from yellow to pink.”*

Adverse effects from the procedure and medications were also described, such as pain, cancer, diabetes, peripheral edema, acne, and weight gain.

#### Lifestyle

The need to remain adherent to a strict regimen of post-transplant medications, follow-up appointments, bloodwork, and limitations on physical activities (e.g., sports) were perceived to impact patient and caregiver lifestyles. Patient from transcript 18 said: *“Being as I was a recent transplant recipient, contact sports were something they wanted me to avoid. I guess I still did sports but not really high contact sports.”*

#### Return to normal

Many participants remarked on how receiving a transplant had allowed the patient and family to return to normal. Transcript 21:

*“They always give you the worst possible outcome, and of course that’s what you focus on. . .We had a couple times where his body didn’t want to cooperate, so we had some setbacks, but for the most part we had all adjusted really well and once he was ready to come home after the transplant, he was just a normal five-year-old kid.”*

#### New life perspective

Some patients and caregivers described how transplantation had changed their life. For example, one patient (transcript 1) mused:

*“I was kind of your nerdy, average kid. . .It’s life changing, obviously, from a health side but from a spiritual and mental side. It reminds you of what’s important and focuses you. It kind of gives you a life goal. . .I technically should not be here. It’s just by happenstance that I had the appointment with the doctor, it’s just by happenstance that we discovered end stage renal disease; the chips all kind of fell in the right spot.”*

### Category 2: Transplant Expectations

Two subcategories emerged around the central category of “transplant expectations.”

#### Transplant as a cure

Some patients and caregivers embarked on their journey believing that receiving a new kidney would cure all ailments and life would go back to normal post-transplant. It was disappointing for them to learn that kidney transplant is another form of kidney replacement therapy, but not a cure for their kidney disease. For example, a caregiver from transcript 15 said,

*“I just thought, once this transplant is over, it’s over. This rollercoaster will be over and we’re going to have our precious little baby and everything’s going to be so good. It really wasn’t that easy. . . It’s like, will it ever get better?”*

#### Unexpected experiences in the transplant process

Many aspects of the transplant process were unexpected. Some caregivers were unprepared for wait times, numerous hospital visits required during pre-transplant evaluation and the reality of post-transplant care. Transcript 11:

*“You need to come back once a month for appointments or once every couple of weeks, or now there’s blood work once a week, once a month, however many times they tell us to do it. I don’t remember them ever telling us about all that stuff.”*

Some spoke of surprising aspects of the surgery, such as the length of the procedure, or what post-operative care consisted of. Transcript 21: *“I don’t think they really told us about all the tubes that would be in and out of [patient], so it was a shock to see him in the ICU as he was waking up.”* Participants also described feeling unprepared for post-transplant side effects, associated co-morbidities and complications, such as diabetes, hypertension, infections or the emotional impact of the transplant process. For example, a caregiver from transcript 10 said, *“So, I would say the psychology side of things is one thing that I wasn’t prepared for.”* A patient (transcript 2) recalled, *“Three weeks before graduating, I had to get a biopsy. And that just created this shitstorm essentially of, ‘This is totally scary, and I don’t know what all these things mean.’”*

### Category 3: Need for Support

Given the significant social, mental, physical, and lifestyle impacts of transplant, the need for support was a common theme.

#### Practical support

For families traveling out of province for a transplant, the initial 6 weeks post-transplant meant a complete separation from their daily routines. Participants described how support networks allowed them to focus on the transplant, while alleviating the demands of daily tasks. One caregiver (transcript 6) said (in reference to the other caregiver), *“He had somebody doing that job for him, and he didn’t have to worry about work at all—which I’m sure many people don’t have that privilege, but that’s very important.”* The financial burden of living away from home for an extended period is considerable. Multiple participants expressed appreciation to their transplant team in making available financial aid resources part of the transplant planning, as well as appreciation for the direct financial support provided by non-profit agencies. Practical support was especially important for caregivers.

#### Mental health support

Many participants valued the mental health support they received through the transplant process. This support came from formal referral to mental health professionals and from informal personal connections. Participants described that their social networks provided essential daily encouragement through the challenges they faced. Transcript 15: *“If you’re having a bad day, you could reach out to them and say, ‘I’m feeling discouraged today or I’m feeling tired.’ I think that would be so important. . .sometimes one just needs a little bit of moral support.”*

#### Healthcare support

Many participants expressed their appreciation for the support of their healthcare team. Families perceived this support in the information and explanations they received. Transcript 9:

*“It was all so well explained, especially when it came to the surgery. The surgeon himself that did it, he came back several times and if he would see that we were uncomfortable, he would just ask us or tell us more.”*

Another demonstration of support was the availability of the healthcare team to answer questions. Transcript 15:

*“I’m a firm believer, if you’re concerned, then you need to reach out. Even if it’s to have them give you reassurance. They were always there and they would tell [primary caregiver] what to do. Like, “Try this or try that.” And the phone was always open.”*

Transcript 14: *“We have so much support there, they’ve always had anything we need or want to discuss.”*

Patients described that the contact with their healthcare team alleviated their concerns and allowed them the freedom to enjoy life post-transplant. Transcript 2:

*“This. . .doctor was like, ‘What’s something you’ve always wanted?’ That was actually how he put it and I was like*, ‘*I want to get my ears pierced.*’ *He’s like, ‘Ok, let’s get your ears pierced.’ And yeah, you think about when you’re told no so many times and then this person who’s now your doctor says yes.”*

Participants expressed the value they found when their healthcare team was working with them toward shared goals. Transcript 10: *“When you ask educated questions you can become part of the team and working as a team for the good of the patient is terrific. The best, it’s the most constructive thing you can do.”*

#### Support from shared-lived experience

Participants who made a strong connection with someone who had been through the transplant process found this to be a valuable support. Transcript 6: *“We actually had a friend that was. . .ahead of us all the time with her process so that was a big bonus for us. It was kind of an answer to the prayer, so we knew. . .what to expect.”* These connections were made both organically during the time patients were at the hospital for care, and when patients specifically sought out this kind of support. Transcript 3:

*“I almost feel like being on dialysis has helped me little because also you meet a lot of people on dialysis that have either had a transplant already or you would meet nurses that had seen people before and after, so there was a lot of different areas to gain information from and different perspectives.”*

#### Finding a community

Communities may be large or small, and looked different for each participant. Both patients and caregivers described the support they felt from being part of a community that embraced them. For one caregiver, this community was the town in which they live. Transcript 15: *“The whole community cheers for her, the whole community is so proud now that they see her out and about.”* For one patient, the community was their classroom at school. Transcript 20: *“I had some really supportive friends in elementary school. I would get large doses of prednisone before I went on dialysis, and I had a friend who would come with me.”* Some communities formed during the transplant process. As one patient described (Transcript 21):

*“I was staying at the [House] for a long time, so I met some friends. . .I have a lot of memories there, just playing around with kids that were kinda like me. It was very nice. I’d definitely say it was pretty helpful. I got out of my comfort zone and know that I’m not alone in this.”*

#### Challenges with support

Despite the necessity of a support network, for some participants, even this was not without challenges. Many described the importance of confirming a support network prior to transplant, but some felt unable to reach out and ask for support. Transcript 15: “*Maybe we should have. . .explained more to other family members. We could have had other family members want to be part of [patient]’s circle; part of her team. That would have helped.*” Keeping a support network informed often came with the burden of information-sharing. Transplant 11: “W*e actually kept a journal and just posted in on Facebook. . .so everybody could see the updates and everything. We didn’t have to tell everybody twenty different times.”* These challenges perpetuated the feeling of social isolation through transplant. Transcript 10: “*Nurses felt like friends, and they were for a while. They were great. But I really did feel quite alone.”*

Several participants described how they were able to rely on their support networks to meet care requirements. Transcript 14:

*“There’s that planning involved in. . .how as a family you can make sure that your appointments are covered. If you and your husband are full-time workers, do you have another support system that can maybe help you get your child to appointments? We are lucky that we had that.”*

However, this wider support network does not usually receive direct education on care tasks from the healthcare team, so training supports becomes an additional role for the primary caregiver(s). Transcript 6:

*“Only me and [second caregiver]. . .touch those medications. If something would pop up and we couldn’t be available. . .it would be very important to actually teach family members a few of those things. Even my sister knows that [patient] needs a lot of fluid, but she doesn’t exactly know the amount of fluid she actually needs.”*

### Objective 2. Characterizing Educational Needs

Participants were encouraged to reflect on information, resources and supports they perceived to be helpful and what could have been done to improve their journey. Their responses revealed key insights.

#### Format and delivery of educational materials

Participants consistently emphasized the importance of accessible and engaging educational formats. Both caregivers and pediatric recipients valued child-friendly resources such as story books, illustrative “road maps” and media such as animated videos. These formats helped make complex medical information more digestible and less intimidating. For example, one patient (Transcript 1) said *“In a perfect world, you make like a Magic School Bus episode. ‘This is your kidney, this is what it does, this is what it means when they fail and these are your options.’”* Caregivers also appreciated structured formats like binders and pamphlets that could be referenced throughout the care journey. Transcript 14: “*We got a booklet of basically everything; questions that you can have, things that can come up, changes that’ll happen within her body.”*

#### Timing and repetition

Participants stressed the importance of receiving information gradually and repeatedly. Education delivered before and after surgery, and reinforced over time, was seen as more effective and less overwhelming.

#### Age-appropriate and emotionally sensitive content

Educational content needed to be tailored to the child’s age and emotional readiness. Younger children benefited from simplified explanations focused on outcomes, while older children required more detailed information as they became more involved in their care. According to one patient, *“At that age, a very simple like, ‘We’re taking this because it makes you feel better.’ That would’ve been sufficient for me.”* (Transcript 2). Caregivers often shielded children from distressing details to avoid unnecessary fear or non-compliance. Transcript 17: *“You don’t tell the kids things like, ‘you’re at high risk for cancer.’”* Transcript 9: *“She asks me, why all of a sudden do I get hair on my hands? I don’t tell her. Because if I would tell her, she won’t be taking that medication anymore.”*

#### Caregiver vs. patient information needs

Caregivers typically sought comprehensive, proactive information to manage care and anticipate complications. They valued open communication with the transplant team and often took the lead in learning and decision-making. For example, one caregiver said, “*We’re constantly pushing for information as opposed to it flowing to us*.” (Transcript 16). Patients, especially younger ones, preferred simplified and immediate information, often relying on caregivers to interpret and filter details.

Transcript 14: “*I usually just ask my mom . . . I’ve never picked up a booklet*.” Transcript 20: “*I let my mom take the lead because it was easier.”*

#### Structure and routine, and knowing what to expect

Establishing predictable routines and clear expectations was particularly important for younger patients. Knowing what to expect helped reduce anxiety and fostered a sense of control. Transcript 2: *“Just having that sense of structure. . . what can we expect coming up.”* Patients explained that even as a child, it’s important to be told what to expect. Those that were provided with sufficient information ahead of time to prepare expressed gratitude for it. According to one patient,

*“In terms of surgery, at that age, I still remember, meeting with a vascular surgeon, they were going over all the different things they were going to do. That actually put my mind at ease a little bit, to be honest.” (Transcript 1).*

#### Support systems and emotional needs

Participants highlighted the value of strong relationships with the transplant team and access to emotional support and that they appreciated the opportunity for open, unrushed conversation with the entire transplant team. For example, one caregiver said, “*If you ask a question to the post-transplant team, they are really good about taking the time to explain it*.” Families expressed a desire to be connected with other providers such as social workers who can help with finding financial support or accommodations, or dietitians and connection to mental health supports to help with the emotional and psychological impacts of transplant was perceived to be of particular importance. One caregiver (Transcript 10) said:

*“So, I spoke with his doctor who gave recommendation to Psychology who joined our team at that point. We found a fantastic counselor. And she was able to bring in this this rather sullen, withdrawn, angry guy. . . she was able to make him feel safe and give him privacy and he was able to say whatever he wanted to say. . .Now it’s two years later, and it’s not like [patient] is not going to be susceptible to low feelings. The thing is now he’s got tools to overcome them.”*

Support preferences varied widely. While some children preferred privacy and family-only support, others saw value in peer connections. *“We offered talking to other people about it. He didn’t want that so we just sort of handled it while we were together as a family.”* (Transcript 17)

*“I think she would have really benefited from having somebody else to talk to.”* (Transcript 20). Among those who sought out peer support, it was perceived to be of great benefit. One patient (Transcript 3) said:

*“I learnt about this group of young adults that had had transplants. . .I definitely would recommend that for future transplant patients to get involved with a group of people that have had transplants, ‘cause there is experiences and questions that only they can answer.”*

Some participants who did not have the opportunity to receive peer support expressed the value this would have brought to their experience with transplant. Transcript 8: *“I think the ones that I would really love is lived experience. If there is a community of people that have done that, and they are able and willing to help others.”*

Table 5. presents resources, tools, and information that participants perceived to be helpful, while Table 6 presents the advice that participants would offer to help other patients and caregivers with their transplant journey. There is an emphasis on the importance of support as well as patience, positive attitude, communicating with the transplant team and asking questions. Patients and families would be well served by programs that work to provide social support, promote patient/family network development in addition to providing comprehensive, age-appropriate education.

**Table 5.** Resources, Tools, and Information That Participants Perceived To Be Helpful.

		Resource	Transcript number
Resources that participants had access to that they perceived to be helpful	Health monitoring	- Medication chart with information on side effects, role, administration information, etc	2,3
		- Medication timer (a pocket timer, app, etc.)	20
		- Pulse oximeter, blood pressure monitor, thermometer	3,9,6
		- G-tube for fluid/medication administration	1
		- Book for documenting all medication adminstration, dialysis start times, tube feeds, flushes, etc	9 9
	Ways of learning	- Binder with important information on medications, signs of rejection, dialysis, etc	3,6,14,17
		- Child-friendly information on medications and side effects to watch out for	18
		- Book for grade school children about post-transplant life	8
		- Tackling transplant learning “piece-meal”	10
		- Yearly review of medication side effects and expectations	16
	Connections to support	- Out-patient family room in the hospital	11
		- Living journal on Facebook to update family and friends	15
		- Contact with animals/support dogs while in hospital	14
		- “Kidney camp”	4
		- Mentorship/peer-support/support groups - Kinsmen Foundation for financial support/accommodation	4,6,8,9,10,11,15,17, 18,20,21
Resources that participants did not have access to that that would have been helpful	Health monitoring	- Mobile/texted lab results	1,20
		- Book to document medication/fluid intake reminders, lifestyle changes, appointments, feelings/emotional challenges, etc	1,2,10,15
	Ways of learning	- Online/mobile resource of binder described above	2,9,10,15,20
		- Cartoon episode on kidney transplant	10
		- Choice of resources to explain transplantation to children (eg, app, book, games)	15
		- Customizable videos explaining transplant to a child suited to that child’s interests	2
		- Book detailing what to expect on the transplant journey for patients or caregivers	2
		- Visual road map of the transplant process	10
		- Documentation of conversations with transplant team in a virtual format for review	15
	Connections to support	- Script for transplanted children to explain transplant to peers	14
		- Pediatric coordinator or guidance program for pediatric patients	6
		- Caregiver supports to help offset the care burden/access to homecare	4

**Table 6.** Advice Participants Provided for Other Patients and Caregivers (in the Participants Own Words).

Quote	Transcript number
“*Establish a support system for the parents. Partnering with maybe their brother or sister or their close friends and actively having a network that understands what they’re gonna be going through, that would have been huge for my parents. . .Trying to take things in stride and normalize things. Maybe some kids don’t want that but if you grow up with it normalized, it just makes that transition so much easier if that’s all you’ve ever known. Yeah, I would say those are the key pieces.”*	Transcript 2
“*The patient should have very good contact with their kidney team*”	Transcript 9
“*There’s ups and downs. Always try to look for the bright side*.”	Transcript 21
“*I think I would say, get your little circle of caregivers, prior. We have the team, they’re going to make sure that [patient]’s okay, but sometimes one just needs a little bit of moral support*.”	Transcript 15
“*Always have things ready, your necessities that you need. Have them as much ready as you can. When you just have to up and leave to the hospital for an emergency, it just gives you peace of mind that you don’t have to run around and chase after [things]*.”	Transcript 9
“*I think to have as positive as an attitude as you can. One, because you’re going to beat yourself up and you’re not going to be healthy to be strong enough for your kid, but yet you also don’t want to put your stresses that you’re stressing on over the situation on your kid so that they see that. I think if a kid sees their parent stress out, they’re gonna stress out. Plus, they already have their own stresses*.”	Transcript 21
“*I guess the important thing to know is [if] you do worry about anything at all like any little thing at all just tell your doctor, because you are not going to settle yourself unless you know for sure*.”	Transcript 6
*“Be strong for yourself and for the family member. So, be strong and have a positive mindset.”*	Transcript 8
*“It’s not forever. It’s very tough—the first month especially. But you just have to think what a huge gift they have gotten. . .You just have to be strong even if you feel terrible inside*”	Transcript 6
*“Trust your doctor but do your research. That would be the baseline advice I could give; Be involved, ask questions, trust your doctor.”*	Transcript 10
*“I think you need a good support team.”*	Transcript 21
*“I guess one of the biggest things would just to be patient. You might not feel 100% when you’ve been transplanted. It might take a little while and there is a lot of information—it can be overwhelming. You only need to remember what you need for that date, because there’s that day and the next day. As you get familiar with that, and you keep asking the information, it will just become part of your life. It won’t be so overwhelming. The first couple weeks definitely are, but there is a lot of support there”*	Transcript 3
*“It’s not going to necessarily be a miracle cure.”*	Transcript 20
*“Just expect some more doctor appointments in the future, okay. Some good, some bad, but it’ll work.”*	Transcript 11
*“Do what they tell you to do. And listen to yourself. When you’re getting tired, rest; do whatever you can. Try to be patient. Ask questions.”*	Transcript 16
*“Once you actually start to feel better physically. . .it’s night and day. Your energy’s going to come back. You can feel it. It’s a tough recovery, but it’s worth it in the end.”*	Transcript 1

## Discussion

Given the importance of education to improve success of pediatric transplantation and the lack of literature in this area, we conducted a study to characterize the experiences of patients and caregivers and to learn more about their educational needs. Previous reports have described the transplant process as stressful or overwhelming^8-13^ and have emphasized the life impacts on both the patient and caregiver.^2,20,21^ In the present study, patients and caregivers discussed how the journey to transplant is all-encompassing, with wide-ranging effects on the family’s social, mental health, and physical well-being.

While both patients and caregivers described the transplant journey as emotionally and logistically challenging, their experiences diverged in focus and perspective. Caregivers often emphasized the stress of managing care responsibilities, navigating healthcare systems, and coping with uncertainty—particularly around donor eligibility, post-operative complications, and long-term outcomes. They also expressed concern for the emotional well-being of their child and the impact on siblings and family dynamics. In contrast, patients reflected more on the personal and social consequences of transplant, such as feeling different from peers, missing out on milestones, and adjusting to lifestyle changes. Patients also described moments of empowerment and resilience, particularly when regaining physical strength or forming connections with others who had similar experiences. These differences underscore the need for holistic support strategies that address both the caregiving burden and the lived experience of the patient.^22^ While patients and caregivers shared overlapping educational priorities, such as the desire for clear expectations and accessible formats, their needs diverged in important ways. Caregivers often sought comprehensive, proactive information to manage care and anticipate complications, preferring structured resources like binders and verbal explanations. In contrast, patients, particularly younger ones, benefited more from simplified, emotionally sensitive education tailored to their developmental stage, often delivered through visual or interactive formats. Caregivers frequently acted as information filters, shielding children from distressing details, while patients tended to rely on caregivers for interpretation or preferred to receive information only when immediately relevant. These differences highlight the importance of designing transplant education that is both age-appropriate and role-specific, ensuring that each member of the care team—patient and caregiver alike—is adequately supported.

Since most patient participants in the present study (70%) were adults reflecting on their childhood experiences, we compared educational insights across age groups within this cohort. Educational needs were shaped more by developmental stage and individual preference than by age alone. Children often relied on caregivers to interpret medical information and preferred simplified, emotionally sensitive explanations, focusing more on living life than understanding clinical details. In contrast, adults—particularly those reflecting on their younger selves—described feeling overwhelmed by complex information and emphasized the value of structure, routine, and visual aids to support comprehension. Both groups appreciated gradual, age-appropriate education, with younger patients benefiting from messages like “this makes you feel better,” and older individuals seeking more detailed, realistic discussions over time. Retrospective accounts highlighted that visual or interactive tools, such as videos or cartoons, could have eased understanding and reduced fear during childhood. These findings underscore the importance of flexible, patient-centered educational strategies that evolve with cognitive and emotional readiness. However, since only three participants in our study were under 19, further research is needed to better understand transplant education from the perspective of younger patients themselves.

As previously reported,^2,7,21^ the need for support was recognized as fundamental by both patients and caregivers, yet obtaining and maintaining a support network was not without obstacles. Education may help mitigate some of the challenges described by patients. Healthcare providers are optimally positioned to provide education that helps patients and caregivers form connections to the supports that they identify. Our study identified several challenges that we feel could be addressed by education. For example, to decrease the explanation burden on primary caregivers, healthcare providers could create a resource suitable for both alternate care providers and for knowledge transfer and sharing with friends and family.

A consistent message that arose within the dialogue was that both patients and caregivers benefit when they know what to expect. Participants recounted experiences where they were surprised by events that occurred before the transplant (such as the wait times and hospital visits), during the transplant (e.g., such as the length of the procedure, or what a child looked like post-operatively), and after the transplant (such as medication side effects, co-morbidities and complications). A previous study of family members’ experiences with patients’ treatment for end-stage kidney disease (including transplant) indicated that the degree of impact that transplant can have on daily lives and mental health can be unanticipated.^21^ Notably, some unexpected experiences were attributed to a lack of forewarning from healthcare providers.^21^ By providing effective education, healthcare providers can support families by helping them anticipate the transplant course more accurately. Participants in the present study described some novel educational ideas to facilitate preparedness, such as a visual road map of the transplant process or customizable video-based resources specific to a child’s interests. This has also been supported by other literature.^9^

Despite the advantages of being ready as events unfold, the healthcare journey is unpredictable, no two experiences are alike, and support needs may vary for each individual throughout the journey. In the present study, frequent sources of support were found in community networks, but this looked different for every participant. Some patients and caregivers relied on extended family and friends, whereas others found support from religious communities. As healthcare providers, we tend to use a standardized approach for providing education and support, but we must recognize that this might not be enough. In the present study, some participants expressed the desire for a more personalized approach and suggested a choice of age-appropriate resources, such as books, video games, and cartoons. The desire for situation-specific information^8^ and a preference for receiving information in a variety of formats^12^ was also identified in our scoping review of pediatric transplant education.^7^ However, creating personalized educational materials is both time and resource intensive and novel solutions can be explored. Our group is presently exploring the use of technology such as ChatGPT to create pediatric patient education. We also advocate for the use of relevant pediatric focused resources and internet-based web applications. For example, Transplant Families is a non-profit organization that offers a virtual space for resources and educational materials to help families throughout the transplant process. (https://www.transplantfamilies.org/).

The limitations of this study should be considered. Participants were recruited from a single center in Canada, and our participants were predominantly White, which limits generalizability. In the present study, we interviewed patients who previously received a transplant and their caregivers. Exploring perceptions of those who are in the pre-transplant stage (i.e., being assessed for transplant or on the waitlist) may garner additional unique insights. We included adults who had received a transplant as a child, and seven of the participants were over the age of 20. For the patient participants, all had received their transplant at least 5 prior to participation in the study and were reflecting on their past experiences in hindsight. Program changes may have occurred since their transplant experience. While these testimonials could be influenced by recall bias, thoughts and emotions recounted years later should still be considered relevant. All interviews with adolescent participants were conducted jointly with their caregivers. Although this was the choice of the participants, conducting paired interviews may influence feedback, as individuals might not speak as candidly about their experiences as they would have if interviewed alone. There is potential selection bias, given our sample may over represent patients and families with positive experiences. Finally, the ability to gain information from a wider age range of pediatric patients would have been ideal and we recognize this as a limitation.

## Conclusion

Patients and caregivers described their experiences with the transplant process. Participants identified the need for personalized, age-appropriate education delivered in digestible formats, with clear expectations, timely reinforcement, and emotional support tailored to both patients and caregivers. Being prepared for the transplant can fundamentally impact the experience. As healthcare providers, we should strive to provide personalized and holistic education to facilitate expectations and prepare patients and caregivers for the journey that lies ahead. The participants’ call for enhanced social support throughout the transplant process should not be ignored. The insights gathered from the study will help inform the development of educational resources for pediatric patients and caregivers.

## Supplemental Material

sj-docx-1-cjk-10.1177_20543581251399080 – Supplemental material for Characterizing the Experiences and Educational Needs of Patients and Caregivers During the Kidney Transplant Process Supplemental material, sj-docx-1-cjk-10.1177_20543581251399080 for Characterizing the Experiences and Educational Needs of Patients and Caregivers During the Kidney Transplant Process by Michelle Ruhl, Ashley Burghall, Brianna Groot, Nicola Rosaasen, Kayla Flood, Keefe Davis, Natasha Minakakis, Jenny Wichart and Holly Mansell in Canadian Journal of Kidney Health and Disease
