# Supplementary material for: Characterizing the Experiences and Educational Needs of Patients and Caregivers During the Kidney Transplant Process
Source: Can J Kidney Health Dis. 2025 Dec 25;12:20543581251399080. doi: 10.1177/20543581251399080 (PMC12745526; doi:10.1177/20543581251399080)
Supplement: sj-docx-1-cjk-10.1177_20543581251399080 – Supplemental material for Characterizing the Experiences and Educational Needs of Patients and Caregivers During the Kidney Transplant Process [file sj-docx-1-cjk-10.1177_20543581251399080.docx]

**Semi-structured Interview Guide**

**Introductory Questions:**

1. What is your relationship to the individual that requires (or has received) a kidney transplant?

*Probes: Are you the primary caregiver?*

1. Where are you/your child on the transplant journey? (Assessment vs listed vs transplanted)?

*Probes: Are you/your child on dialysis? Please describe*

*Are you/your child waitlisted to receive kidney transplant?*

*How long have been anticipating that you or your child would need a kidney transplant?*

*Has someone close to you ever had a kidney transplant before*?

If previous transplant: *How long ago was your/your child’s transplant and how has it changed your life?*

**Exploratory Questions:**

1. How well-informed do (or did) you feel about the transplant process?
2. Usually patients begin learning about transplant in advance of their initial discussion with their physician. What information would have been most helpful to you before your transplant discussion with your physician? Please tell me more about that.
3. What information do you think is important to know for transplant caregivers or patients to know about **before the transplant surgery**? Please tell me more about that.

*Probes: About the work up process?*

*About the source of the transplant kidney – living donor vs. deceased donor, increased infectious-risk donors*

*About the travel logistics (e.g. travel time accomodations, stay in Edmonton)*

*About the support needed?*

*About surgery?*

*About care in hospital after transplant surgery?*

*About your medications?*

*About complications/things that can go wrong after transplant?*

*About care in Saskatchewan the first year after transplant, including frequency of bloodwork and visits with the Nephrology team?*

1. What information do you think is important to know **at the time of surgery** and while in the hospital after the operation? Please tell me more about that.

*Probes: About your medications? Fluid requirements?*

*About the follow-up you will need?*

*About complications?*

*About how to assess your own health/symptoms of concern?*

*About changes that you might experience in your body?(e.g. energy, appetite, taste)*

*About going back to work and other normal activities?*

1. What information do you think is important to know **before discharge**? Please tell me more about that.

*Probes: About the operation?*

*About the surgery?*

*About the medicines that you will take?*

*About the time you will be in hospital?*

*About the follow-up that you will require?*

*About the complications after the transplant?*

*About returning to normal activities?*

1. What information do you think is important for transplant caregivers or patients to know **when you return home**?

*Probes: About the medications?*

*About the medical follow-up that is required?*

*About the lifestyle changes that are required?*

*About returning to normal activities?*

*About the complications of transplant?*

1. Can you describe how the information you need to know changes over time?

*Probes: Does this change the longer you are from transplant?*

*Do you need reminders about transplant information?*

*About what?*

*If so, how often?*

1. What aspects of the transplant teaching did you like or dislike?
2. Thinking back to what you knew before this experience, is there anything that you know now but would have liked to have been told before?

*Probes: Did you get the right information at the right time?*

*Was anything a surprise to you after transplant?*

*Did you experience anything that you were not told about?*

*Did you find the information overwhelming?*

*What information would have been most helpful to you (at various time points)?*

*What, if any, of the information you received scared/reassured you?*

*Are there education items that would be better conveyed in a different format or at a different time?*

*What was the most confusing part of receiving a kidney transplant?*

1. As part of the transplant journey, children and teens learn more about their transplant and the care required over time. They will gain a larger role in management of their own health until they are independent. When (at what age) would you/your child have wanted to learn more about aspects of the transplant procedure and care that is required afterwards?

*Probes: About the transplant itself?*

*About the medications?*

*About how to assess your own health/symptoms of concern?*

*About complications/things that can go wrong after transplant? (This includes discussions with the team about serious complications like cancer and rejection of the transplant kidney.)*

*About ongoing health care required (e.g., monthly labs, visits q3months with the transplant team)*

*About recommendations for healthy active living, (e.g. discussions around exercise, diet, sexual health, use of alcohol, smoking, vaping)*

*About transition to adult care?*

1. How did you discuss transplant with your child/teen at home, outside of your discussions with the medical team?

*What topics did they want to discuss?*

*What things did they have questions about?*

*Did you feel you had the right resources to explain transplant or any related care to your child?*

*What resources would have helped you in having these conversations?*

1. Were there any burdens/challenges/stressors do you/your family wish you prepared for differently / knew about? If so, please describe.
2. How has the transplant process affected you mentally and emotionally?
3. Are there any supports that you/your family benefited from? Are there any supports that weren’t available that you could have benefited from? If so, please describe.

*Did you feel that your support network understood what you were going through with transplant?*

*What information do you wish they had known to support you better?*

1. If you had to tell another patient or caregiver or family how to prepare for this process, what would be the key things important for them to know?
2. How did you and your family learn about the transplant process? Are there other ways that you would have preferred to receive this information?

examples: *In-person discussion with the healthcare team*

*- Group learning*

*Written handouts*

*Illustrated book*

*Apps or /games*

*Online information*

*Video series*

**Exit Question:**

1. Is there anything else that you would like to tell us that you think is important?

**Demographic information to collect (if not obtained from the interview questions above):**

**These questions will be asked directly to the participant who consents to the interview (child or caregiver):**

**ADULT INTERVIEWEE DEMOGRAPHIC QUESTIONS**

**If the participant is a caregiver:**

Age:

Age of child:

How long have you been a caregiver for a child with kidney disease:

Stage of transplant journey: (e.g. considering transplant, waiting for a transplant, received a transplant)

Province of residence:

Rural or Urban:

Sex:

How would you describe your gender: (male, female, gender diverse)

Marital status:

Education:

How would you describe your ethnicity:

**CHILD INTERVIEWEE DEMOGRAPHIC QUESTIONS**

**If the participant is a child (questions will be tailored to maturity level of participant and some may be omitted depending on the situation):**

Age:

How long have you had kidney disease:

Stage of transplant journey: (e.g. considering transplant, waiting for a transplant, received a transplant)

Province of residence:

Rural or Urban:

Sex:

How would you describe your gender: (male, female, gender diverse)

Education:

How would you describe your ethnicity:
